# PELICAN: A quality of life instrument for childhood asthma: Study Protocol of two Randomized Controlled Trials in Primary and Specialized Care in the Netherlands

**DOI:** 10.1186/1471-2431-12-137

**Published:** 2012-08-30

**Authors:** Stephanie van Bragt, Lisette van den Bemt, Bart Thoonen, Chris van Weel, Peter Merkus, Tjard Schermer

**Affiliations:** 1 Department of Primary and Community Care, Radboud University Nijmegen Medical Centre, The Netherlands; 2 Department of Paediatric Pulmonology, Radboud University Nijmegen Medical Centre, The Netherlands

**Keywords:** Asthma, Quality of life, Children, Primary care, Specialized care, Self-management, Randomized controlled trial (RCT)

## Abstract

**Background:**

Asthma is one of the major chronic health problems in children in the Netherlands. The Pelican is a paediatric asthma-related quality of life instrument for children with asthma from 6–11 years old, which is suitable for clinical practice in primary and specialized care. Based on this instrument, we developed a self-management treatment to improve asthma-related quality of life. The Pelican intervention will be investigated in different health care settings. Results of intervention studies are often extrapolated to other health care settings than originally investigated. Because of differences in organization, disease severity, patient characteristics and care provision between health care settings, extrapolating research results could lead to unnecessary health costs without the desired health care achievements. Therefore, interventions have to be investigated in different health care settings when possible. This study is an example of an intervention study in different health care settings. In this article, we will present the study protocol of the Pelican study in primary and specialized care.

**Method/design:**

This study consists of two randomized controlled trials to assess the effectiveness of the Pelican intervention in primary and specialized care. The trial in primary care is a multilevel design with 170 children with asthma in 16 general practices. All children in one general practices are allocated to the same treatment group. The trial in specialized care is a multicentre trial with 100 children with asthma. Children in one outpatient clinic are randomly allocated to the intervention or usual care group. In both trials, children will visit the care provider four times during a follow-up of nine months. This study is registered and ethically approved.

**Discussion:**

This article describes the study protocol of the Pelican study in different health care settings. If the Pelican intervention proves to be effective and efficient, implementation in primary and specialized care for paediatric asthma in the Netherlands will be recommended.

**Trial registration:**

This study is registered by clinicaltrial.gov (NCT01109745)

## Background

Asthma [1] is the most common chronic disease in childhood in the Netherlands. Its prevalence ranges from 3% in children aged 5–9 years to 3.7% in children aged 10–14 years [2,3]. Although the prevalence is leveling off [4], asthma remains a significant burden for the child, family and the society at large [5]. In the Netherlands, children with intermittent and mild asthma are usually treated by a family physician, while patients with more severe or uncontrolled asthma are treated by specialized paediatric care [6]. This implies that children with asthma treated in primary and specialized care may differ in features like disease severity and complexity, level of symptom control, functional status and co-morbidity [7]. Recent reports point to substantial room for improvement in the management of childhood asthma [8-11]. Poor adherence to therapy and inadequately treatment are two important reasons why asthma is uncontrolled [9]. Poor adherence has been associated with discrepancies between the perceived relevance of treatment goals between patients and their healthcare providers. Usual care for children with asthma focuses on reducing morbidity and mortality by maintaining optimal asthma control, while asthmatic children want to live a normal life with as few limitations from their asthma as possible [12]. Taking the child’s perception into account in the management of its asthma could result in achieving treatment goals of patients and providers and contribute to patient centered care. However, recent studies pointed out that input of children and their parents into asthma management is only taken into account by health care providers during a minority (6 to 10%) of medical visits [13].

One of the important treatment outcomes showing a patient’s perspective is health related quality of life (HRQL). HRQL is a complex of all aspects of an individual’s subjective experience that relate to health, disease, disability and impairment, which is usually measured in physical, emotional, cognitive and social dimensions [14-17]. A HRQL questionnaire adds significant information about the functional impairments of a patient to clinical and physiological characteristics. Previous studies have shown that the most important HRQL components for children are consequences of asthma on peer relationships, dependence on medication, shortness of breath, cough, limitations in activities and limitations due to tobacco smoke exposure [18,19].

We have previously developed a child friendly web based tool to assess asthma-related quality of life in paediatric asthma patients, using the so-called ‘Pelican instrument’. In this paper, we describe the rationale for, and the designs of two randomized controlled trials (RCTs) that are based on the implementation of the Pelican instrument. In these trials the effectiveness of its implementation will be evaluated in both primary and specialized care setting in the Netherlands. Both trials are optimally adjusted in methodological design to meet the needs of the concerned care setting. Because of the known differences in healthcare organization and patient characteristics, it is important to establish possible effects of the treatment in both healthcare settings separately. In the past, results from specialized care studies have often been extrapolated into primary care, and vice versa [20,21]. Knowing that several important differences exist between these care setting, it is likely that this might lead to ineffective, unnecessary or expensive health care without the desired health achievements. Therefore, it is crucial to evaluate such interventions in primary as well as in specialized care.

### Hypothesis

The primary hypothesis of the Pelican study is that a self management treatment based on HRQL information (Pelican instrument) is able to improve HRQL on the PAQLQ-S (> 0.5 points) in children with asthma from 6–11 years old in primary and specialized care with a proportional difference in favour of intervention relative to usual care of 25% of children. Furthermore, it is also hypothesized that the instrument could improve asthma control and satisfaction with care of parents.

### Research questions

#### The primary research question

– Is the Pelican intervention in primary and specialized health care effective in improving HRQL of children with asthma?

#### The secondary research questions

– Is the Pelican intervention in primary and specialized health care effective in improving HRQL of the parents of children with asthma?

– Is the Pelican intervention in primary and specialized health care effective in improving asthma control of children with asthma?

– Does the Pelican intervention improve patient-doctor relationship and satisfaction with delivered care in primary and specialized health care?

– Is the Pelican instrument able to detect and improve psychosocial problems?

– Is the Pelican intervention user friendly in regular medical care and cost-effective in a primary and specialized care setting?

## Methods/design

### Study design

This study consists of two RCTs: one in primary care and one in specialized care. Because the study design, sample size calculation and treatment allocation differ between the trials, these aspects are described separately. All other aspects apply to both trials.

170 participants in primary care will be recruited from 16 general practices in the Netherlands. The primary care study is a multicentre parallel group study with a hierarchical or nested design. This means that all participating patients within a general practices will be allocated to the same treatment group (usual care or intervention group). During visits the usual care group receives care of the GP according NHG (Dutch GP Association) guidelines [22]. Besides usual care, the intervention group will receive recommendations based on the Pelican outcome by a practice nurse as well.

The most optimal study design for intervention studies is a multilevel design to avoid contamination between usual care and the intervention, which is applied in the primary care trial. There are, however, not enough outpatient clinics in the Netherlands to be able to use a multilevel design in specialized care. This means that specialized nurses provide care for both patients of the intervention and those of the usual care group. 100 participants in specialized care will be recruited in 5 outpatient clinics in the Netherlands. The usual care group receives care according to NVK (Dutch Association of paediatric) guidelines of paediatric asthma in the Netherlands of a care provider (e.g. paediatrician or nurse) [23]. The intervention group receives an intervention with the Pelican outcome by an asthma nurse complementary to usual care.

### Sample size calculation

A multilevel power calculation was performed for the trial in primary care based on the percentage of children with an improvement of 0.5 points on the PAQLQ instrument score (Δ0.5 is the minimum clinically important difference (MCID) for the PAQLQ) [14,24]. We consider a proportional difference in favour of intervention relative to usual care of 25% of children with a MCID to be clinically relevant. Based on the assumptions ICC = 0.04, α = 0.05, 1-β = 0.80, 20% of usual care group with a 0.5 points increase in PAQLQ score, and dropout rate of 15%, a total of 170 children with asthma needs to be included from 16 GPs.

A power calculation was performed for the trial in specialized care based on the percentage of children with an improvement of 0.5 points on the PAQLQ instrument score. We consider a proportional difference in favour of intervention relative to usual care of 25% of children with a MCID to be clinically relevant. Based on the assumptions α = 0.05, 1-β = 0.80, 10% of usual care group with a 0.5 points increase in PAQLQ score, and dropout rate = 15%, a total of 100 children with asthma needs to be included.

### Selection phase

In both trials children aged 6 to 12 years with asthma diagnosed by a physician will be recruited. Moreover, children must have used their asthma medication (i.e., bronchodilators and / or inhaled corticosteroids) for at least six weeks during the previous year to confirm ‘active asthma’. Exclusion criteria for these studies are: 1) a comorbide condition that significantly influences the HRQL, 2) not being able to attend a regular school class and 3) insufficient skill of Dutch language.

Parents of children are informed about the study by verbal explanation and an information brochure. For children, age-adjusted study information is provided on the website of the study. Written informed consent of both official caregivers will be obtained to ensure voluntary and anonymous participation. In accordance with the guidelines on scientific research with underage individuals, children may be withdrawn from the study at any time if they object [25].

### Randomisation: treatment allocation

Enrolment of participants will be performed by the general practices or outpatient clinics. Participants can be allocated to the usual care or intervention group. In both trials, the minimization technique will be used to allocate participants to treatment. Minimisation is an advanced randomisation technique as described by Pocock [26,27]. The balance between both groups will be kept with consideration of prognostic factors (categorical variables) as described below with a computer program called Minim.

In the primary care trial general practices will be allocated balanced on the following prognostic factors: 1) number of potential participants for study within GP (<18 or ≥18 patients with the diagnosis asthma between 6–11 years old) and 2) usual asthma care (absence of structured asthma care, structured asthma care for adults or structured asthma care for children).

In the specialized care trial the minimisation technique will be used to force treatment allocation of individual participants with consideration of the prognostic factors age (6–8 years old and 9–11 years old) and level of asthma control (ACQ score <1 and ACQ score ≥ 1).

### Pelican instrument

The paediatric Electronic quality of Life Instrument for Asthmatic children in the Netherlands or Pelican is a web-based asthma-related quality of life questionnaire that is presented as a web-based computer game. It is effortless to complete, easy to understand (questions are read aloud) and full of attractive sounds and pictures. While playing the game, the child answers questions about his or her experience of asthma. Item selection for the Pelican was based on children’s personal perspectives that were obtained using focus groups [18]. Items are concrete aspects of day-to-day life with asthma what makes them an easy starting point for treatment and are scored in burden experience on a 5-point Likert scale ranging from ‘no burden at all’ (0 points) to ‘very high burden’ (5 points). Scores are visually supported by emoticons. Besides overall quality of life, the instrument has the ability to priorities issues of asthma bothering the patient most.

### Intervention

The intervention is a self-management treatment based on the patient’s reply to the Pelican questionnaire. Treatment is provided by a specialized nurse with the objective to improve the patient’s asthma-related quality of life. The nurse is trained during a 2-hour meeting in this intervention with theoretic background, video instructions and role playing exercises. Supplementary to the training, a minimum of five evaluation moments are included during the trial.

The child fills out the Pelican before the scheduled visit and the outcome is forwarded to the nurse. The intervention is based on the theory of behaviour change and shared decision making [28]. The health care provider supports the parent and child to explore their needs and treatment goals (according SMART principles [29]) and think about solutions for disease related problems and challenges. Together, the parent, child and nurse work on a treatment plan to improve disease management resulting in better disease outcomes. The treatment plan is a mutual agreement that is specific, acceptable and realistic. During the next visit, the nurse will evaluate the effectiveness of the treatment plan: why was it successful or unsuccessful to achieve treatment goals. If not successful, the care provider will explore the causes (lack of knowledge/skills, motivational, practical or social barriers) and evaluate whether they can be resolved.

### Study parameters

The primary outcome of the study is HRQL as measured with the PAQLQ-S (standardised activities). The Dutch version of the PAQLQ was validated in children ≥ 6 years and has psychometric properties similar to those reported for the original PAQLQ [30]. Also the HRQL of the main caregiver is assessed with the PACQLQ (paediatric asthma caregiver quality of life questionnaire) [31].

Secondary outcomes are asthma control, psychosocial problems and satisfaction with care. Asthma control is measured by questionnaires such as the Asthma Control Questionnaire (ACQ) [32], the Childhood Asthma Control Test (C-ACT) [33] and the Asthma Therapy Assessment Questionnaire (ATAQ) [34]. Psychometric properties of all asthma control instruments were found to be good [32,33,35]. Lung function with reversibility (pre- en post- FVC, pre- and post- FEV1) and Fraction of exhaled NO^1^ (FeNO) will be assessed according international guidelines [36,37]. Psychosocial problems will be measured with the Dutch version of Strengths and Difficulties Questionnaire (SDQ) that shows good psychometric properties [24]. An adapted version of the Patient-Doctor relationship Questionnaire (PDRQ-9) will be used to measure parent’s satisfaction with provided care to their child by a care provider and other patient-doctor relationship aspects [34].

### Data collection

In the recruitment phase, the parents receive a questionnaire on family characteristics (e.g., socio-economic status, family history of respiratory diseases, composition of family, smoking habits), child asthmatic symptoms, medication use, attitude towards asthma [35]. All participants visit the general practices or outpatient clinic four times during a follow-up period of 9 months. The parents as well as the children fill out a questionnaire at the start and end of the study to measure HRQL, asthma control, psychosocial problems and satisfaction with care. During the first visit, lung function will be measured of subjects in the primary care trial. In specialized care, both lung function and fraction exhaled NO will be measured during the first and last visit. Next, the parents fill out monthly calendars on asthma control, symptoms and medication use of their child. Finally, health care providers, parents and children will be inquired about the satisfaction and user friendliness of implementation of the Pelican using structured questionnaires. Since the Pelican study is a complex intervention, data will be collected for process evaluation.

### Data analysis

The primary analysis is an intention-to-treat analysis, however both explanatory and intention-to-treat analyses will be performed. The effect of the Pelican in primary care will be analysed using multi-level analyses techniques. The effect of the Pelican in specialized care will be assessed using multivariate regression models. A correction for baseline value of the outcome of interest (e.g., HRQL, asthma control) will be added to the model.

### Process evaluation and cost-effectiveness analysis

The implementation of the Pelican in health care is a complex intervention [38]. A detailed process evaluation is crucial to evaluate what the active ingredients are and how they are exerting their effect. Potential barriers and facilitators of implementation are screened in every phase of the study, starting at the completion of the Pelican instrument by the child at home, the use of the Pelican instrument during the medical visit by the health care professional and finally the influence on asthma specific HRQL [39].

A cost-effectiveness analysis (CEA) from a societal perspective will be performed and reported according to national and international guidelines [40,41]. The costs included will be program costs, direct medical costs and indirect costs measured in their natural units and transformed to costs using real prices and standard reimbursement of expense tariffs. An effect is defined as a relevant change in HRQL (i.e., ΔPAQLQ ≥0.5 point) in the denominator and the total of all relevant costs in the nominator.

### Ethics

Ethical approval is obtained from the Medical Ethics Committee of the Arnhem-Nijmegen region in the Netherlands. The study protocol is extensively studied by: The Dutch Asthma Foundation, NutsOhra and Radboud University Nijmegen Medical Centre (RUNMC). This trial is registered by clinicaltrial.gov (NCT01109745).

## Discussion

Patient centred care, especially in chronic diseases, has a growing need to implement information on health related quality of life (HRQL) next to clinical and physiological measures. Although several quality of life instruments for paediatric asthma patients already exist, the Pelican instrument distinguishes itself because of its applicability in regular medical care. In this article, we presented the methodological design of the Pelican study. Our primary research question is whether the use of the Pelican will improve the asthma-related quality of life in children with asthma from 6–11 years old. The secondary research questions are whether the use of the Pelican intervention will improve asthma control, satisfaction with care and HRQL of the parent. Furthermore, it will be evaluated whether the Pelican is able to detect and reduce psychosocial problems. The study consists of two RCTs with a follow-up of nine months performed in primary and specialized care. Although the study design, sample size and treatment allocation of participants differ between both trials, the research questions and data collection will be identical. The trial in primary care will be a multilevel design, while participants within outpatient clinics of specialized care will be randomly allocated to the usual care and intervention group.

The Pelican study has a couple of strong characteristics. In this study, we choose to add another HRQL instrument to the data collection besides the Pelican instrument as an independent HRQL measurement to answer our primary research question. Another strong characteristic of this study is that the intervention will be investigated in both principal health care settings for childhood asthma, doing justice to the differences that exist in organisation and patient characteristic between primary and specialized care. For example, paediatric asthma patients in primary care show lower disease severity compared to patients in specialized care [7]. This could imply different levels in room for improvement in HRQL and thus could lead to different effect results. Performing two trials which are methodologically optimised to the health care setting leads to better validity and reliable results of treatment effect and cost effectiveness analyses. Furthermore, a detailed process evaluation will be done to map barriers and facilitators of the treatment.

However, some drawbacks must be mentioned. It is impossible to perform a double-blind RCT because patients will be aware of the treatment group allocation. In addition, being a subject in an intervention study will cause the Hawthorne effect, easily leading to a favourable response [42-44]. A specific limitation of the primary care trial is the frequency of medical visits. To keep up scientific comparability between the two trials and actively work on a treatment plan with the patient, the frequency of four medical visits during a period of nine months was set. This frequency of medical visits is usual in specialized care but doubled the frequency of visits in primary care compared to the recommendations in national guidelines. Usual care in the primary care study is, therefore, best described as ‘enhanced usual care’ [45]. Increasing contact time between child and care provider may give more favourable results. This effect is expected to be equal in both study arms in the primary care trial. Ideally, a usual care group without any protocol enhancements would have been added to the RCTs. Furthermore, we choose to let the Pelican intervention be performed by a practice nurse. Nurses play an increasing important role in health care for chronic patients, such as diabetes and COPD patients. Involvement of paediatric nurses is already part of usual care in most paediatric outpatient clinics while paediatric asthma management in primary care is usually provided by family physicians without involvement of nurses. A recent study of Kuethe et al. (2011) suggested that the level of asthma control in children managed by an asthma nurse is not inferior to traditional management by primary or specialized care physicians [46]. Our choice of practice nurse involvement was made with the eye on expected future changes in paediatric asthma care in family practices.

A limitation in the specialized care trial is the risk on contamination bias. The specialized nurses in outpatient clinics provide care for patients of the intervention and usual care group. Although nurses were instructed not to use intervention techniques in the usual care group, contamination bias is unavoidable. The trained nurse in outpatient clinics might unintentionally apply training aspects (such as shared decision-making) during treatment of patients in the usual care group and may potentially minimize the difference in outcomes between the two treatment groups.

In conclusion, the aim of this study is to assess the effectiveness of the Pelican in primary and specialized care through a self-management treatment with the aim of improving asthma-related quality of life in children with asthma from 6–11 years old. If the Pelican proves to be effective and efficient, implementation of this instrument in usual care for paediatric asthma will be recommended. Although the implementation of the Pelican intervention will be evaluated in two almost equal RCTs, it was necessary to adjust the design of both trials to fit the concerning settings. Although, different study design characteristics lead to less comparability between the two trials, the unique feature of this project is that we do not need to extrapolate study outcomes to another care settings based on numerous assumptions, as the intervention is evaluated simultaneously in both major care settings.

## Abbreviations

ACQ: Asthma control questionnaire; ATAQ: Asthma therapy assessment questionnaire; ACT: Asthma control test; C-ACT: Childhood asthma control test; FeNO: Fraction exhaled nitric oxide; FEV1: Forced expiratory volume in one second; FVC: Forced vital capacity; GP: Family physician/practice; HRQL: Health related quality of life; PACQLQ: Paediatric asthma caregiver quality of life questionnaire; PAQLQ: Paediatric asthma quality of life questionnaire; Pelican: Paediatric electronic quality of life instrument for childhood asthma instrument in the Netherlands; PDRQ-9: Patient doctor relationship questionnaire; SDQ: Strengths and difficulties questionnaire; UMC: University medical centre.

## Competing interests

The authors declare that they have no competing interests.

## Authors’ contributions

SvB, LvdB and TS have developed the protocol. LvdB, TS and BT applied for funding. SvB drafted the manuscript with critical input from all authors. All authors read and approved the final manuscript.

## Pre-publication history

The pre-publication history for this paper can be accessed here:

http://www.biomedcentral.com/1471-2431/12/137/prepub
